# Femoral rotation and medial flexion gap tightness in functional alignment robotic total knee arthroplasty for varus knees

**DOI:** 10.1007/s11701-026-04037-6

**Published:** 2026-10-12

**Authors:** Eduardo Frois Temponi, Pedro Henrique Oliveira Gomes, Alexandre Leite de Andrade, Miguel Jourdain Alípio do Vale, Matheus Braga Jacques Soares, Luiz Fernando Machado Soares, Lúcio Honório de Carvalho Júnior

**Affiliations:** 1 Department of Medicine, Pontifical Catholic University of Minas Gerais (PUC Minas), Belo Horizonte, Brazil; 2 Grupo Joelho, Serviço de Ortopedia, Hospital Madre Teresa, Avenida Raja Gabaglia, Belo Horizonte, Minas Gerais 1002 Brazil

**Keywords:** Arthroplasty, Replacement, Knee, Robotic Surgical Procedures, Osteoarthritis, Knee, Medial collateral ligament, Knee, Rotation, Range of motion, Articular

## Abstract

Functional-alignment (FA) robotic total knee arthroplasty (TKA) combines component positioning and soft-tissue management. This study described the association between medial flexion-gap tightness and surgeon-selected femoral rotation, together with release grades and intraoperative gap asymmetry in varus knees. This observational single-surgeon study included the 65 varus knees in an 89-knee database. Medial tightness was defined as lateral minus medial preoperative flexion gap. Correlation and ordinary least-squares regression used 63 complete observations. Recorded preoperative, planned and final trial gaps were analysed with endpoint-specific denominators. An exploratory model considered tibial coronal positioning and posterior slope. Selected rotation was associated with medial tightness (Spearman ρ = 0.571; p < 0.001). The univariable fit was rotation = 1.93° + 0.343°/mm × tightness (R² = 0.257; slope 95% CI, 0.194–0.492). The adjusted slope was 0.296°/mm (95% CI, 0.131–0.460; n = 60). Release was mild in 64/65 and moderate in 1/65 knees. Mean absolute final asymmetry was 0.38 mm in extension and 0.29 mm in flexion. Asymmetry <1 mm occurred in 50/60 (83.3%) and 55/59 (93.2%), respectively; 53/58 (91.4%) met ≤1 mm in both positions. Greater medial flexion tightness was associated with greater selected femoral rotation. Predominantly mild release and small final mean asymmetry accompanied the combined workflow. The equation describes one surgeon’s decisions and is not a validated surgical rule. These observations do not establish an independent release-sparing effect, clinical superiority of submillimetre symmetry, patellofemoral safety or long-term benefit.

## Introduction

Functional alignment (FA) individualises component positioning with reference to anatomy and soft-tissue balance. Constitutional varus and variation in femoral and tibial morphology challenge a uniform alignment target [1, 2]. Robotic planning supports individualised component positioning [3], but an identical medial–lateral gap at every flexion angle should not be equated with native laxity [4].

The varus knee frequently has a tight medial compartment. Stepwise medial soft-tissue management can address imbalance but requires care to avoid excessive release [5, 6]. Component-position changes also affect gaps [7]. Femoral rotation is therefore one of several coupled adjustments available to the surgeon; an observational relationship between rotation and tightness cannot isolate its independent contribution.

Within an established gap-guided FA workflow, we sought to describe the quantitative association between preoperative medial flexion-gap tightness and femoral external rotation actually selected by the surgeon, and to report the accompanying release grades and final gap asymmetry. The present analysis overlaps with a previously published gap-phenotype derivation study [8] and is exploratory. It was not designed to validate a surgical dosing rule or establish the clinical superiority of submillimetre symmetry.

## Materials and methods

### Study population and relationship to previous work

This observational study analysed prospectively recorded operative data from a single surgeon at a tertiary referral centre, with reporting guided by STROBE [9]. Institutional ethics approval and informed consent were obtained. The analysis included all 65 records classified as varus in an 89-knee primary FA robotic TKA database; 23 valgus and one neutral knee were excluded. The unit of analysis was the knee. Database provenance identifies an initial 68-case cohort and 21 subsequently appended surgical records. Fifty-one of the 65 varus knees belonged to the initial cohort used for the previous derivation study [8], and 14 came from the appended records. Thus, 51 knee records overlap and 14 are additional to that cohort. The 51 linked records represented 51 identifiable patients, with surgery dates from 25 March 2024 to 10 July 2025. Identifiers and dates were unavailable for the 14 appended records; a total unique-patient count and bilateral status across the entire series could not be established. We therefore do not assert verified consecutive recruitment for the complete 65-knee dataset.

The prior study developed a driver-based gap-phenotype classification [8]. The incremental analysis here concerns the rotation–tightness relationship, its adjustment for recorded tibial positioning, and release grades in the varus subset. Gap, alignment and PROM observations from the initial cohort are reused descriptive data, not independent validation or additional follow-up. No new PROM observations were available from the 14 appended varus knees.

### Surgical strategy and measurement states

Procedures used the CT-based MAKO robotic platform and a medial parapatellar approach. Implant designs were recorded as cruciate-substituting (CS), cruciate-retaining (CR) or posterior-stabilised (PS). The operating surgeon confirmed that the posterior cruciate ligament (PCL) was preserved in CS and CR knees and sacrificed in PS knees. Specific implant models, software version and timing of PCL management relative to gap acquisition were not available for this analysis. Medial and lateral gaps were recorded under distraction in extension and at 90° flexion. The preoperative state represented the registered arthritic knee before the release/tensioning interval described in the workflow; the planned state was the recorded finalised virtual component plan, and the final state followed bone cuts and trial insertion. Soft-tissue management occurred between preoperative and planned recordings. The records did not establish whether the planned gaps incorporated repeat laxity acquisition after release or retained the initial acquisition. Planned-state values therefore represent a recorded workflow state and do not isolate virtual positioning from soft-tissue changes.

Component positioning was gap-guided within the FA workflow. The available records contained selected femoral rotation, tibial coronal positioning and posterior slope, but not the registered femoral rotational reference axis, prospectively permitted limits, fixed-versus-native slope selection criteria, or the chronology of individual adjustments. Positive rotation was interpreted in the original operative record as external rotation; this convention was retained without assigning an undocumented anatomical reference axis. Observed ranges are reported as observed values, not reconstructed protocol boundaries. Tibial varus and slope are the recorded planning parameters. Intermediate slope changes, anatomical individualisation criteria and the exact sequence of tibial adjustments, releases and femoral rotation could not be reconstructed. Distraction loads, calibration details and patellar position were also unavailable, limiting reproducibility.

Medial management was graded by the operating surgeon according to the recorded extent (Table 2). Mild release comprised osteophyte removal and limited subperiosteal elevation of the deep medial collateral ligament (MCL) and posterior oblique ligament, preserving the superficial MCL. Moderate release included additional posteromedial capsular or semimembranosus release or controlled needle puncturing; major release involved the superficial MCL or more extensive pie-crusting [5, 6]. These were descriptive surgeon-assigned categories rather than independently validated thresholds. The previous numerical escalation rule was not retained as a validated algorithm. No posterior capsulotomy was performed. Interobserver and intraobserver reliability were not assessed.

### Variable definitions and statistical analysis

**Fig. 1:**
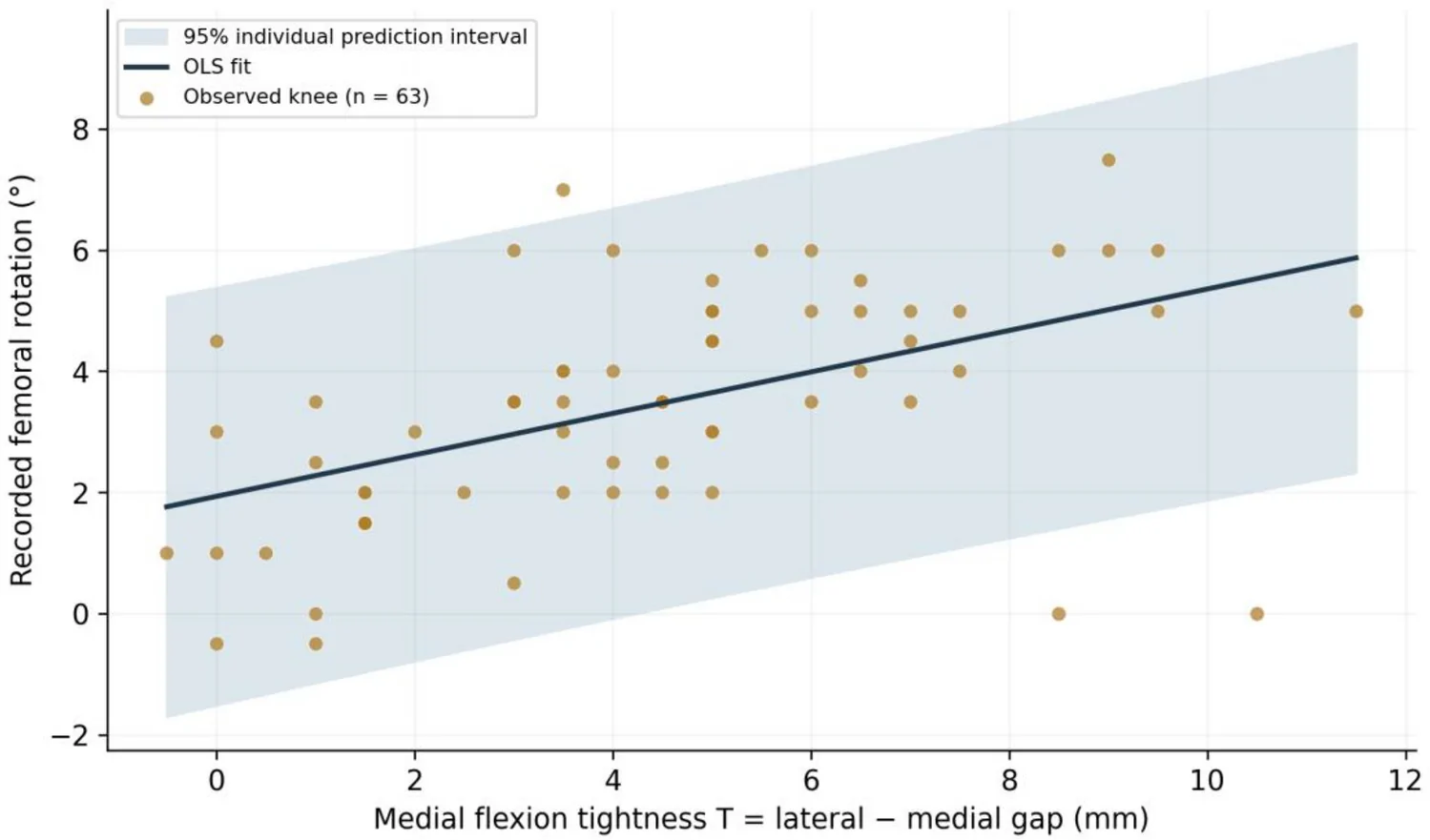
Observed association between medial flexion tightness T (lateral minus medial gap) and selected femoral rotation in 63 knees. Points are actual paired observations; coincident points may overlap. The line is the ordinary least-squares fit (rotation = 1.933° + 0.343°/mm × T; R^2^ = 0.257); shading is the conventional 95% individual prediction interval. Negative T values were retained. The reference axis could not be recovered. Neither the fit nor the interval defines a surgical target or safe rotational range

The signed gap difference was D = medial gap − lateral gap (mm). Medial flexion tightness was T = lateral gap − medial gap = −D, so positive T indicates medial tightness. Negative T was retained and indicates relative medial laxity; T was neither made absolute nor truncated at zero. Absolute asymmetry was |D|. Correlation, regression and Fig. 1 used the same T variable and complete observations. Final strict gap symmetry was defined as |D| ≤1 mm in both extension and flexion in the same knee. Position-specific submillimetre rates used |D| <1 mm. A measurement exactly at 1 mm satisfies only the inclusive threshold. Coronal alignment was evaluated separately against the original prespecified HKA envelope of ±3° from neutral. The database’s “functional balance” flag was not analysed because its quantitative definition could not be recovered.

Continuous variables were summarised as mean ± sample standard deviation; counts use their assessable denominators. All gap differences were checked against the compartment measurements. Spearman correlation and ordinary least-squares regression described the relationship between T and selected rotation. Conventional two-sided 95% confidence intervals and individual prediction intervals were calculated. A post hoc exploratory model added tibial varus and posterior slope as recorded covariates, chosen for their potential relevance to balancing rather than selected by statistical significance. Because only two PS knees were present, implant/PCL effects were not estimated; a sensitivity analysis repeated this model in CS/CR knees with preserved PCL. These models describe operative decisions, not independent causal effects. Paired Wilcoxon signed-rank tests compared preoperative and final absolute gaps. Available cases were used for each outcome, complete cases for each regression, and matched observations for paired analyses; no data were imputed. Calculations used Python with NumPy and SciPy. All additional analyses were exploratory, without multiplicity adjustment.

WOMAC [10] and FJS-12 [11] were secondary descriptive outcomes, using the recorded preoperative and day-365 total scores on 0–100 scales (lower WOMAC and higher FJS-12 indicate better status). Item-level responses and scoring/missing-item rules were unavailable for audit. Availability was reported against all 65 knees and the 51-record linked subgroup. Exact assessment dates, eligibility for the one-year visit and reasons for missing scores were unavailable; these proportions describe data completeness rather than a verified scheduled-visit retention rate. Paired WOMAC change included only records with both time points. Range-of-motion endpoints, radiographic OA grades, MPTA and LDFA were not available. Recorded native and final sagittal angles describe intraoperative extension posture and must not be interpreted as maximum flexion or clinical range of motion.

## Results

### Cohort and operative characteristics

The cohort comprised 65 varus knees (Table 1). Age was available for 50 knees (73.9 ± 6.6 years); sex was available for 51, including 40 females (78.4%). These replace the earlier demographic summaries drawn from the broader linked cohort. Native varus HKA averaged 7.28 ± 3.61° (n = 65). Varus severity was <5° in 18/65 (27.7%), 5° to <10° in 31/65 (47.7%) and ≥10° in 16/65 (24.6%). Reducibility, calculated as native minus corrected absolute HKA, averaged 5.89 ± 2.72° (n = 61); 31/61 (50.8%) had a corrected HKA of 0°, while 30/61 retained residual varus. Designs were CS in 58, CR in five and PS in two knees; the PCL was preserved in 63/65 (96.9%) and sacrificed in 2/65 (3.1%).

**Table 1:** Cohort characteristics and recorded positioning

Characteristic	Available result
Cohort / earlier cohort overlap	65 varus knees; 51 overlapping and 14 additional knee records
Linked demographics	Sex: n = 51; female 40/51 (78.4%). Age: n = 50
Age, years	73.95 ± 6.62; range 56.0–90.6
Native HKA, degrees varus	7.28 ± 3.61; range 0.5–18.0; n = 65
HKA reducibility, degrees	5.89 ± 2.72; range 0.5–14.0; n = 61
Native sagittal angle, degrees	6.38 ± 6.28; range −7 to 27; n = 65
Final sagittal angle, degrees	2.17 ± 1.56; range −2 to 7; n = 63
Femoral rotation, degrees	3.44 ± 1.92; range −0.5 to 7.5; n = 64
Recorded tibial varus, degrees	1.01 ± 1.01; range 0–3; n = 62
Recorded posterior slope, degrees	2.90 ± 0.48; range 0–3; n = 64
Implant design / PCL	CS 58, CR 5: PCL preserved. PS 2: PCL sacrificed
Final HKA, degrees varus	2.28 ± 1.68; range −2.5 to 5.5; n = 64

Values are mean ± sample SD unless stated otherwise. Positive HKA denotes varus; negative final HKA denotes valgus. Negative sagittal angle denotes hyperextension. Sagittal angles are intraoperative extension-posture measurements, not clinical ROM. Observed component ranges are not protocol limits. CS, cruciate-substituting; CR, cruciate-retaining; PS, posterior-stabilised; PCL, posterior cruciate ligament.

Recorded tibial varus averaged 1.01 ± 1.01° (n = 62; range, 0–3°), and posterior slope averaged 2.90 ± 0.48° (n = 64; range, 0–3°). Slope was 3° in 61/64 knees. It averaged 2.92 ± 0.44° in CS knees (n = 57; range, 0–3°), 3.00 ± 0.00° in CR knees (n = 5), and 2.00 ± 1.41° in PS knees (n = 2; observed values, 1° and 3°). These observations do not establish a fixed slope protocol or design-specific selection rule.

### Femoral rotation and medial flexion tightness

Selected femoral rotation averaged 3.44 ± 1.92° (n = 64; range, −0.5° to 7.5°). The association used 63 knees: one lacked rotation and a different knee lacked preoperative flexion gaps. T ranged from −0.5 to 11.5 mm. Spearman correlation was +0.571 for T (p <0.001), equivalent to −0.571 for D on the same observations. The recalculated univariable equation was rotation = 1.933° + 0.343°/mm × T (R^2^ = 0.257). The intercept 95% CI was 1.151–2.715° and the slope 95% CI was 0.194–0.492°/mm (Fig. 1).

At T = 5 mm, predicted rotation was 3.65° with a 95% individual prediction interval of 0.26–7.03°. The interval represents statistical uncertainty, not a safe rotational range. In the exploratory model including T, tibial varus and posterior slope (n = 60), the T coefficient was 0.296°/mm (95% CI, 0.131–0.460; p = 0.0007; model R^2^ = 0.274). The corresponding tibial-varus coefficient was 0.355°/° (95% CI, −0.106 to 0.815), and the slope coefficient was 0.087°/° (95% CI, −0.814 to 0.988). Restriction to PCL-preserved CS/CR knees (n = 58) yielded a T coefficient of 0.279°/mm (95% CI, 0.106–0.453; R^2^ = 0.274). The narrow slope distribution and sparse PS subgroup limit adjustment; unmeasured anatomy and sequencing remain potential sources of confounding.

### Release and technical balance

**Fig. 2:**
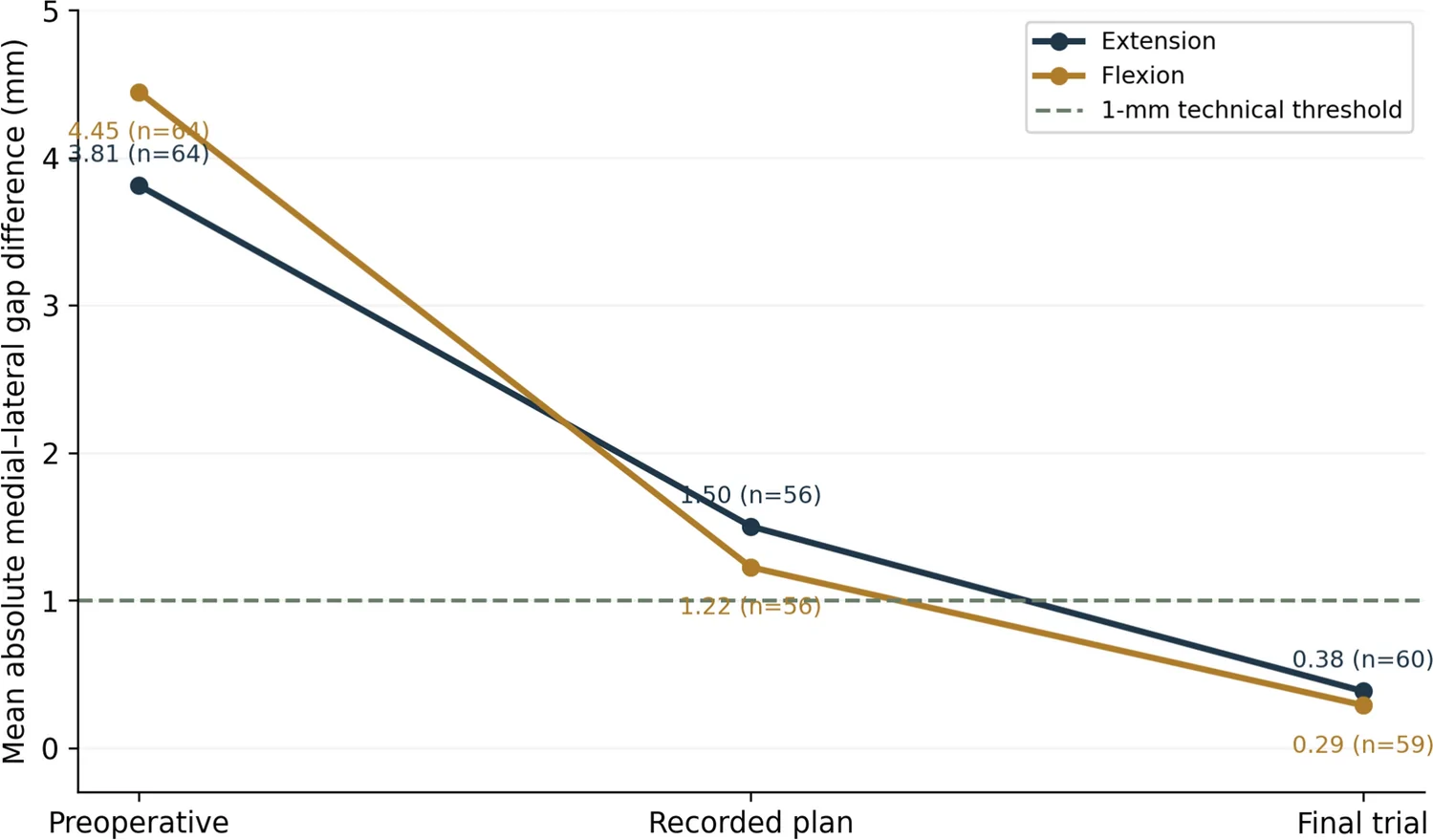
Mean absolute gap asymmetry at each recorded state, with available-case denominators. State-specific means include different assessable sets and should not be read as a complete-case trajectory. Paired preoperative-to-final comparisons used 59 extension and 58 flexion observations. The 1-mm line is a technical threshold, not a proven clinical optimum. Intervening soft-tissue management precludes attributing the preoperative-to-planned change to virtual planning alone

Release was mild in 64/65 knees (98.5%), moderate in 1/65 (1.5%), and major in none (Table 2). Mean absolute extension asymmetry was 3.81 mm preoperatively (n = 64), 1.50 mm at the planned state (n = 56) and 0.38 mm at final trial (n = 60). Corresponding flexion values were 4.45 (n = 64), 1.22 (n = 56) and 0.29 mm (n = 59; Fig. 2). Preoperative-to-final paired comparisons were significant in extension (n = 59) and flexion (n = 58; both p <0.001). State-specific plotted means use available observations and are not a single complete-case trajectory. The reductions accompanied component adjustments and soft-tissue management and cannot be attributed to planning or rotation alone.

**Table 2:** Graded medial soft-tissue management

Grade	Recorded extent	Count
Mild	Osteophyte removal and limited deep-MCL/posterior-oblique elevation; superficial MCL preserved	64/65 (98.5%)
Moderate	Additional posteromedial/semimembranosus release or controlled needle puncturing	1/65 (1.5%)
Major	Superficial-MCL release or extensive pie-crusting	0/65 (0%)

Categories were assigned by the operating surgeon; reliability was not assessed. The categories describe recorded practice and do not validate escalation thresholds.

Final |D| <1 mm was achieved in 50/60 knees in extension (83.3%) and 55/59 in flexion (93.2%). Final |D| ≤1 mm was achieved in 56/60 (93.3%) and 57/59 (96.6%), respectively. Among 58 knees with both positions recorded, 53/58 (91.4%) met the inclusive threshold in both (Table 3). Thus, the original percentages described different thresholds and assessable populations. Final HKA averaged 2.28 ± 1.68° varus (n = 64; range, 2.5° valgus to 5.5° varus); 46/64 (71.9%) were within ±3°. The remaining 18/64 (28.1%) were >3° varus, reaching 5.5°. Reasons for exceeding the envelope were not documented.

**Table 3:** Final technical balance and alignment

Outcome	Criterion	n/N (%)	Missing /65
Extension submillimetre	|D extension| <1 mm	50/60 (83.3)	5
Flexion submillimetre	|D flexion| <1 mm	55/59 (93.2)	6
Extension inclusive	|D extension| ≤1 mm	56/60 (93.3)	5
Flexion inclusive	|D flexion| ≤1 mm	57/59 (96.6)	6
Simultaneous strict symmetry	Both positions |D| ≤1 mm	53/58 (91.4)	7
HKA within envelope	−3° ≤ HKA ≤3°	46/64 (71.9)	1

D = medial minus lateral gap. Six extension and two flexion observations were exactly 1 mm in absolute magnitude. Position-specific denominators differ because measurements were missing. The undefined functional-balance flag is omitted.

Baseline WOMAC was available for 40/65 knees (61.5%), and day-365 WOMAC and FJS-12 for 25/65 (38.5%) each, equivalent to 25/51 (49.0%) of linked records. Baseline WOMAC averaged 74.61 ± 23.46; day-365 WOMAC averaged 2.50 ± 4.46 and FJS-12 95.25 ± 15.65 (Online Resource 1). In the 21 matched records, WOMAC decreased from 72.97 ± 24.53 to 2.98 ± 4.73 (mean reduction, 69.99 ± 25.29; paired p <0.001). At day 365, 18/25 had WOMAC = 0 and 21/25 had FJS-12 = 100, indicating marked floor/ceiling concentration. These limited available-case outcomes cannot be generalised to all knees or attributed to gap symmetry.

## Discussion

In this single-surgeon series, greater preoperative medial flexion tightness was associated with greater selected femoral external rotation. Predominantly mild release and small final mean gap asymmetry accompanied the combined workflow. Because the surgeon used gap information to choose component positions, the association is partly built into the decision process. These observations do not show that rotation independently reduced releases or improved patient outcomes.

**Fig. 3:**
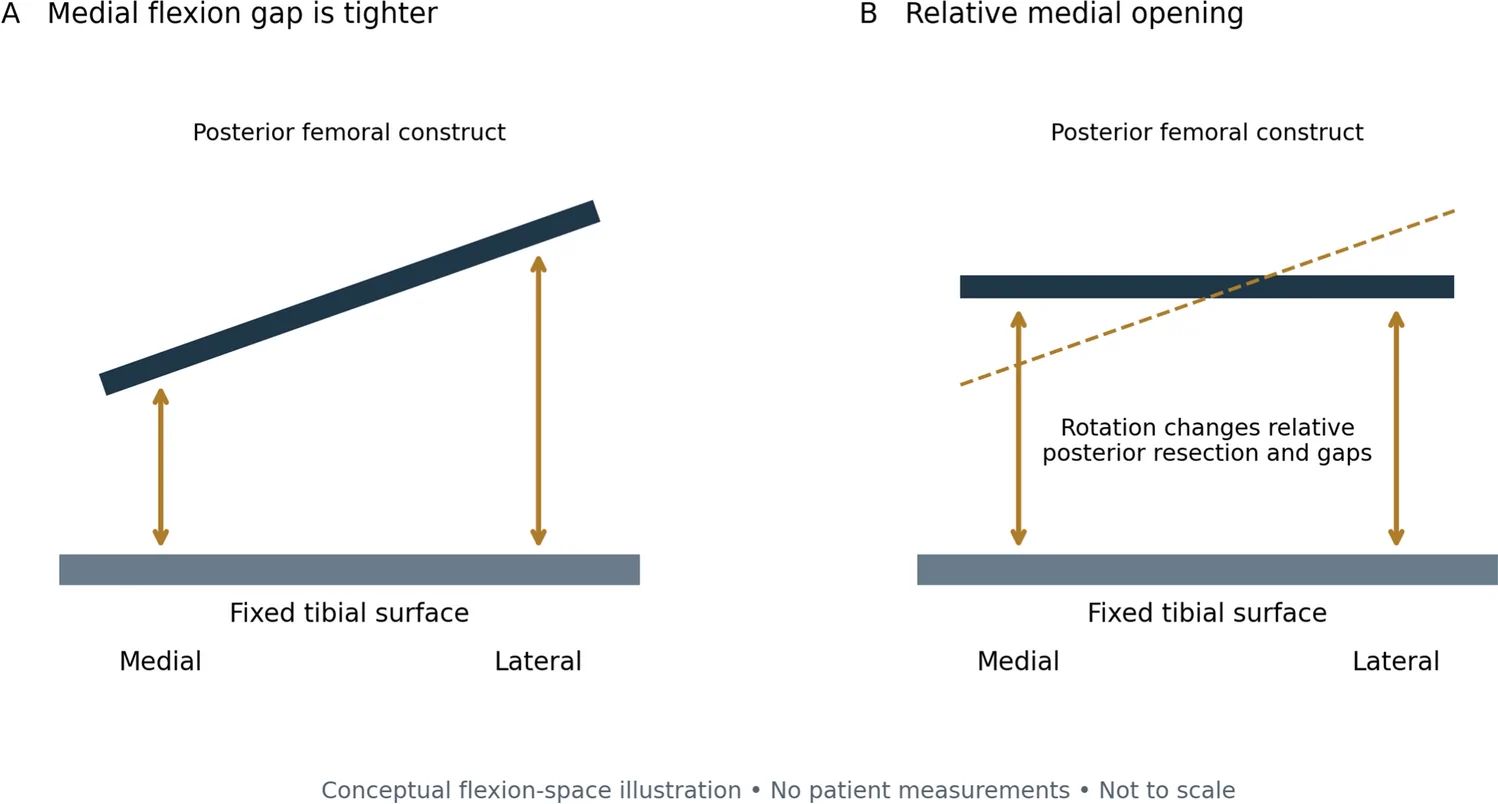
Conceptual flexion-space illustration. A, medial tightness relative to the lateral side. B, femoral rotation changes relative posterior resection and can increase medial clearance relative to lateral clearance with a fixed tibial surface. The dashed line shows the initial construct. Actual effects depend on the pivot, size, translation, slope and soft-tissue state. No patient measurements are depicted; the schematic is not to scale and is not a validated operative algorithm

External rotation can change the relative posterior femoral resection and increase the medial flexion space relative to the lateral space, depending on the pivot, component size, translation and referencing convention (Fig. 3). Tibial positioning and soft-tissue tension also shape the flexion space [7]. Posterior slope and PCL management can alter flexion gaps [12, 13], so a rotation-only interpretation is not justified. Similarly, a medial release should not be assumed to act exclusively in extension; its effects depend on the structures released and the flexion angle [5].

The fitted equation is an exploratory description of surgeon-selected rotation, not a generalisable titration rule. The univariable R^2^ of 0.257 and wide prediction interval indicate substantial unexplained variation. The association persisted after including recorded tibial varus and slope and in the PCL-preserved subgroup, but model fit remained modest. These analyses do not address unmeasured anatomy, the rotational reference axis, timing of releases or coupled component adjustments. Slope was nearly constant and PS knees were too few for a meaningful PCL comparison. External validation with prospectively defined measurements is required before any decision aid is considered.

The decrease in asymmetry between preoperative and planned states reflects a workflow interval that included component adjustments and soft-tissue management. It is inappropriate to attribute most correction to virtual planning alone. Final trial gaps are an intraoperative technical assessment, not proof of dynamic stability, weight-bearing radiographic balance or patient-perceived function. Eighteen of 64 knees (28.1%) fell outside the prespecified ±3° HKA envelope, all in residual varus. These are target outliers; their undocumented rationale cannot be recast as intentional constitutional preservation. The uncontrolled design cannot determine their clinical implications.

The 1-mm criterion was a technical symmetry target, not a demonstrated optimum for clinical outcomes. Applying the same threshold in extension and flexion simplifies reporting but does not reproduce all aspects of native knee laxity. Native laxity varies with flexion [4], and modelling of robotic balancing has allowed greater lateral laxity in flexion than extension [7]. This series did not compare different symmetry targets or establish a relationship between |D| <1 mm and WOMAC or FJS-12. Accordingly, greater physiological lateral flexion laxity may be acceptable, and universal submillimetre symmetry should not be inferred to be clinically superior.

Patellofemoral effects require a separate safety assessment. External rotation is not uniformly harmful: one intraoperative clinical study reported lower patellofemoral contact stress with greater external rotation within its studied conditions [14]. Conversely, biomechanical work shows that rotation away from the epicondylar reference can adversely affect tracking and tibiofemoral wear-related motion [15]. These findings do not establish a universal safe angle or prove that greater rotation inevitably worsens tracking. The effects depend on the reference axis, anatomy, component design and combined positioning. Patellar tracking, tilt, anterior knee pain and patellofemoral radiographic measures were not systematically recorded here, so the patellofemoral consequences of the observed strategy cannot be quantified. The regression must not be used to justify large rotational changes without an independent anatomical and patellofemoral assessment.

### Limitations

This uncontrolled, single-centre, single-surgeon series cannot establish causation or reproducibility across surgeons, platforms, designs or populations. Fifty-one knee records overlap with the derivation cohort, so the findings are not an independent validation. The 14 appended records lacked patient identifiers and dates, preventing complete patient-level deduplication, confirmation of consecutive recruitment and ascertainment of follow-up eligibility. Missing clinical ROM, OA grade, MPTA and LDFA limit anatomical and clinical characterisation. The rotational reference axis, protocol boundaries, detailed operative sequence and gap-loading conditions could not be reconstructed, and the planned state did not separate virtual changes from soft-tissue management. The rotation association is partly design-driven; limited covariate adjustment does not establish an independent effect on releases or outcomes. Release grading lacked reliability testing, and only one moderate release occurred. Distracted gaps do not equate to loaded laxity, dynamic kinematics or patient perception. Variable measurement completeness and precision constrain submillimetre comparisons. Only 25/65 knees had day-365 PROMs; unknown missingness mechanisms, unaudited item-level scoring and marked floor/ceiling concentration limit interpretation. No clinical superiority of strict symmetry was tested. Patellar tracking, tilt, anterior knee pain and patellofemoral radiographic measures were not systematically assessed. One-year PROMs cannot establish long-term wear, aseptic loosening, revision risk, implant survival or late failure.

## Conclusion

In 65 varus knees treated by one surgeon using FA robotic TKA, greater medial flexion tightness was associated with greater selected femoral rotation. Predominantly mild release and small final mean gap asymmetry accompanied the combined workflow. The equation remains exploratory despite limited covariate adjustment. These data do not establish an independent release-sparing effect of rotation, clinical superiority of submillimetre symmetry, patellofemoral safety or long-term implant survival.

## Data Availability

No datasets were generated or analysed during the current study.

## Declarations

### Relationship to prior work

Part of the authors’ ongoing research line in robotic knee arthroplasty and functional alignment; advances their prior published work in this area (Temponi et al., J Robot Surg 2026) with a larger, varus-focused series addressing a distinct, practice-oriented question.
